# Thermosensitive Biodegradable Hydrogels for Local and Controlled Cerebral Delivery of Proteins: MRI-Based Monitoring of *In Vitro* and *In Vivo* Protein Release

**DOI:** 10.1021/acsbiomaterials.2c01224

**Published:** 2023-01-22

**Authors:** Pavel Yanev, Geralda A.F. van Tilborg, Kristel W. M. Boere, Ann M. Stowe, Annette van der Toorn, Max A. Viergever, Wim E. Hennink, Tina Vermonden, Rick M. Dijkhuizen

**Affiliations:** † Biomedical MR Imaging and Spectroscopy Group, Center for Image Sciences, University Medical Center Utrecht and Utrecht University, Utrecht3584 CX, The Netherlands; ‡ Department of Pharmaceutics, Utrecht Institute for Pharmaceutical Sciences, University Utrecht, Utrecht3584 CG, The Netherlands; § Department of Neurology, University of Kentucky, Lexington, Kentucky40506, United States

**Keywords:** in situ hydrogel, contrast MRI, drug delivery, sustained release, protein release, IVIVR

## Abstract

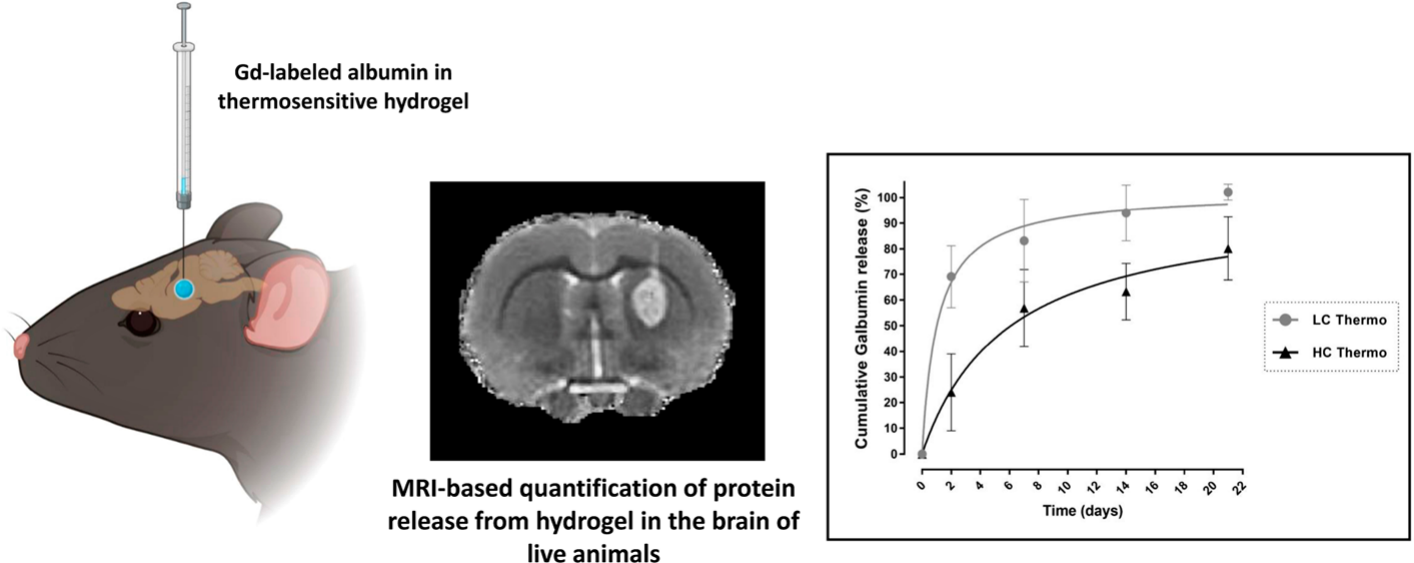

Hydrogels have been suggested as novel drug delivery systems for sustained release of therapeutic proteins in various neurological disorders. The main advantage these systems offer is the controlled, prolonged exposure to a therapeutically effective dose of the released drug after a single intracerebral injection. Characterization of controlled release of therapeutics from a hydrogel is generally performed *in vitro*, as current methods do not allow for *in vivo* measurements of spatiotemporal distribution and release kinetics of a loaded protein. Importantly, the *in vivo* environment introduces many additional variables and factors that cannot be effectively simulated under *in vitro* conditions. To address this, in the present contribution, we developed a noninvasive *in vivo* magnetic resonance imaging (MRI) method to monitor local protein release from two injected hydrogels of the same chemical composition but different initial water contents. We designed a biodegradable hydrogel formulation composed of low and high concentration thermosensitive polymer and thiolated hyaluronic acid, which is liquid at room temperature and forms a gel due to a combination of physical and chemical cross-linking upon injection at 37 °C. The *in vivo* protein release kinetics from these gels were assessed by MRI analysis utilizing a model protein labeled with an MR contrast agent, i.e. gadolinium-labeled albumin (74 kDa). As proof of principle, the release kinetics of the hydrogels were first measured with MRI *in vitro*. Subsequently, the protein loaded hydrogels were administered in male Wistar rat brains and the release *in vivo* was monitored for 21 days. *In vitro*, the thermosensitive hydrogels with an initial water content of 81 and 66% released 64 ± 3% and 43 ± 3% of the protein loading, respectively, during the first 6 days at 37 °C. These differences were even more profound *in vivo*, where the thermosensitive hydrogels released 83 ± 16% and 57 ± 15% of the protein load, respectively, 1 week postinjection. Measurement of volume changes of the gels over time showed that the thermosensitive gel with the higher polymer concentration increased more than 4-fold in size *in vivo* after 3 weeks, which was substantially different from the *in vitro* behavior where a volume change of 35% was observed. Our study demonstrates the potential of MRI to noninvasively monitor *in vivo* intracerebral protein release from a locally administered in situ forming hydrogel, which could aid in the development and optimization of such drug delivery systems for brain disorders.

## Introduction

The blood-brain barrier (BBB), part of the vasculature of the central nervous system, is a gateway for selective entry and provides a physical barrier that inhibits the passage of various molecules, including pharmaceutical proteins to the brain.^1^ Circulating molecules in the blood enter the brain or cerebrospinal fluid (CSF) through the endothelial cells or choroid plexus via several mechanisms: paracellular transportation (small hydrophilic substances), transcellular passive diffusion (small lipophilic substances), solute carrier (SLC) transporters present in the BBB (polar hydrophilic small molecules like amino acids and carbohydrates), and receptor-mediated transporter endocytosis (macromolecular biomolecules such as insulin, transferrin).^2−4^ Effective treatment of neurological disorders, such as brain tumors and neurodegenerative diseases, depends on the successful delivery of therapeutically active proteins to the brain.^5^

The concentration of intravenously administered proteins is relatively high directly after injection, which significantly increases the risk of toxicity. Moreover, therapeutic proteins with hydrodynamic radii below the renal filtration thresholds (*M*_w_ < 30 kDa) are mostly rapidly eliminated from the circulation.^6^ Thus, frequent injections are required to obtain therapeutic effects, which often result in undesired side effects.^7^ To bypass the BBB and to avoid the unfavorable pharmacokinetic properties of many pharmaceutical proteins after intravenous administration, these drugs can be injected intracerebrally to directly reach their target site in the brain with minimal systemic exposure and toxicity.^8^ However, this invasive approach typically creates a temporary local maximum drug level at the site of administration that decreases in time due to diffusion resulting in subtherapeutic drug levels after a certain time postadministration. To achieve satisfactory long-term treatment response, therapeutic effective tissue concentrations of pharmaceutical proteins should ideally be maintained for prolonged periods of time. For this, the active protein needs to be repeatedly administered in the local (intracerebral) target zone or delivered through a cannula inserted into the brain tissue. These approaches are not only impractical for clinical applications, but also increase the risk of high peak concentrations, possibly causing detrimental neurotoxic effects on brain tissue.^9^ This is particularly relevant for cerebral pathologies like stroke, where tissue surrounding the primary lesion can undergo secondary neurodegenerative changes over prolonged periods of time.^10,11^

A strategy for obtaining therapeutically effective drug concentrations in the brain for prolonged times is the use of implantable biomaterials with controlled polymer degradation that allows for radial diffusion of the loaded drug.^12−14^ Such implants can be based on hydrogels that are under investigation for various biomedical applications, including regenerative medicine and controlled drug release.^15−17^ These three-dimensional networks of hydrophilic polymers absorb and maintain large amounts of water, which create possibilities for neural tissue engineering by providing a way to recreate the extracellular matrix (ECM).^18,19^ The versatile nature of hydrogels also allows for drug/protein loading and tailored release depending on diffusivity of the loaded protein in the hydrogel matrix. This in turn depends on the size of the protein and the mesh size of the hydrogel network, e.g., its cross-link density.^20^ The latter, however, can change in time due to swelling and degradation of the matrix. Thus, local cerebral injection of a hydrogel loaded with a drug/protein may bypass the BBB and create a local depot for controlled release.

In the past decades, stimuli-sensitive hydrogels, which are responsive to environmental stimuli such as chemical substances, temperature, pH, pressure, electric field, etc., have gained increasing attention.^21,22^ Among the stimuli-sensitive hydrogels, *in situ* gel-forming hydrogels are attractive drug delivery systems for treatment of injuries and diseases of the central nervous system (CNS). These gels are in a liquid state before administration but form a three-dimensional structure after injection into, for example, the brain.^23,24^ Polymers exhibiting lower critical solution temperature (LCST) behavior with their cloud point preferably between room and body temperature are soluble in water at low temperature, and self-assemble at elevated temperatures, making them suitable for the design of thermosensitive hydrogels.^25^ As many pathological conditions affecting the brain require prolonged protection against ongoing injurious events or enduring promotion of recovery processes, hydrogel-based drug delivery systems are an attractive approach for local controlled release of therapeutic doses of protective and trophic factors. The effectiveness of the three-dimensional porous structure of thermosensitive hydrogels to load and release drugs and growth factors in the central nervous system (CNS) for tissue regeneration has been investigated in a number of studies.^26−29^

The potential of a drug delivery system depends on the precise tailoring of the design and release properties, in order to sustain the presence of the therapeutic compound at the site of administration, while at the same time maintaining biocompatibility. Thus, adequate measurement and knowledge of release kinetics is a crucial step toward clinical translation. Conventional *in vitro* analyses of drug release, particularly protein-loaded hydrogels, often involve placing samples of the material under near sink conditions, i.e., phosphate-buffered saline (PBS) on top of the gel and withdrawing samples from the release medium at different time intervals, followed by replacement with fresh PBS conditions.^30^ Subsequently, the drug concentration in the withdrawn release samples is determined by Ultra Performance Liquid Chromatography (UPLC)^31^ or UV–visible spectrophotometry.^32^ Also, more advanced approaches, such as fluorescence recovery after photobleaching, have been implemented to study the mobility of proteins in hydrogel matrices.^33,34^ These methods are, however, only suitable for characterization of protein release under well-defined *in vitro* conditions. Thus, there is a need for methods that allow the evaluation of protein drug delivery from locally injected depots in a noninvasive way.

Magnetic resonance imaging (MRI) is a versatile imaging modality featured by excellent soft-tissue contrast and no constraints on imaging depth, which can offer the opportunity for *in vivo* measurement of the distribution or release of locally delivered drugs, as well as their effects on tissue structure and function.^35,36^ For example, local drug release after systemic administration of thermosensitive liposomes loaded with a drug-MRI contrast agent complex has been successfully evaluated in tumor-bearing mice, where transient MRI contrast enhancement after focused hyperthermia provided information on the location and timing of triggered content release.^37^ Besides monitoring drug release, MRI can also be applied to detect changes in hydrogel volume, e.g. due to variation in environmental conditions, such as temperature, pH, light, electric field, and pressure,^38^ or as a result of degradation and subsequent swelling of hydrogel material.^39^

The objective of the present study was to establish an MRI method for *in vivo* monitoring of hydrogel characteristics, protein release and possible tissue responses following intracerebral injection of hydrogels. To this end, we injected two thermosensitive hydrogels of the same chemical composition but of different initial water content^31^ in the brain of rats. Albumin, which has a molecular weight (66 kDa)^40^ comparable to many growth factors that are currently under investigation for treatment of CNS diseases, e.g. angiopoietins,^41^ was chosen as the model protein. To enable serial MRI-based release monitoring, albumin labeled with the paramagnetic contrast agent gadolinium, i.e. gadolinium-labeled albumin (74 kDa), was loaded in the hydrogels. Importantly, hydrogel volume changes and tissue status were monitored with structural MRI.

## Materials and Methods

### 1.1 Hydrogel Formation

**Scheme 1:**
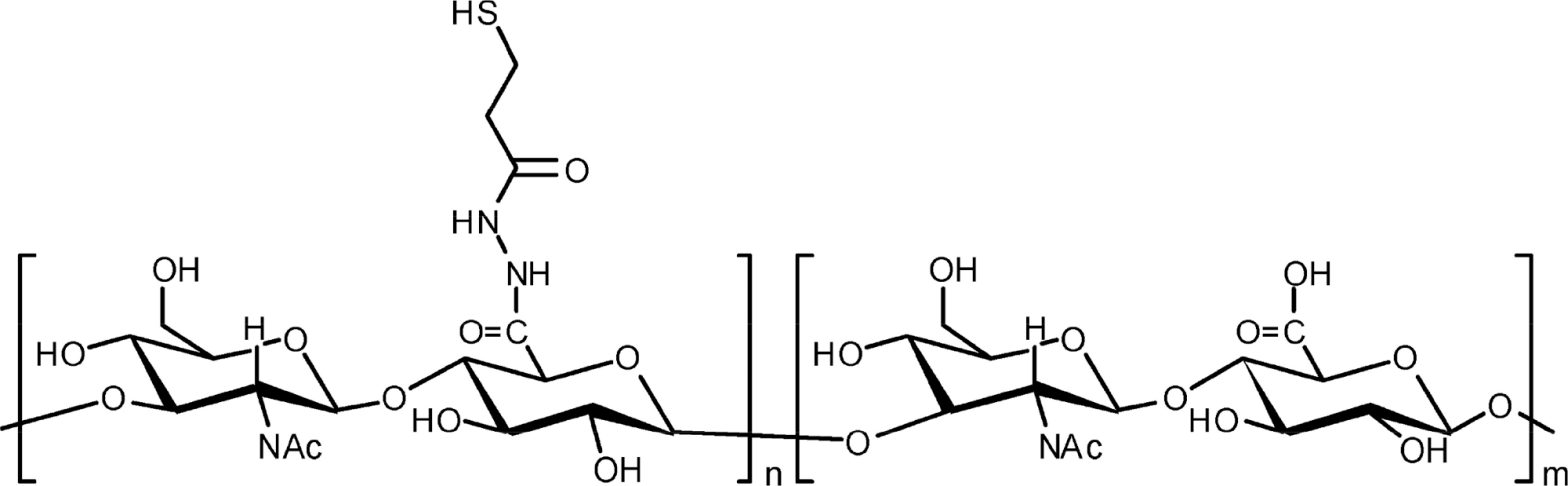
Chemical Structure of Partially Thiolated Hyaluronic Acid (HA-SH) The degree of thiolation was 60%.

**Scheme 2:**
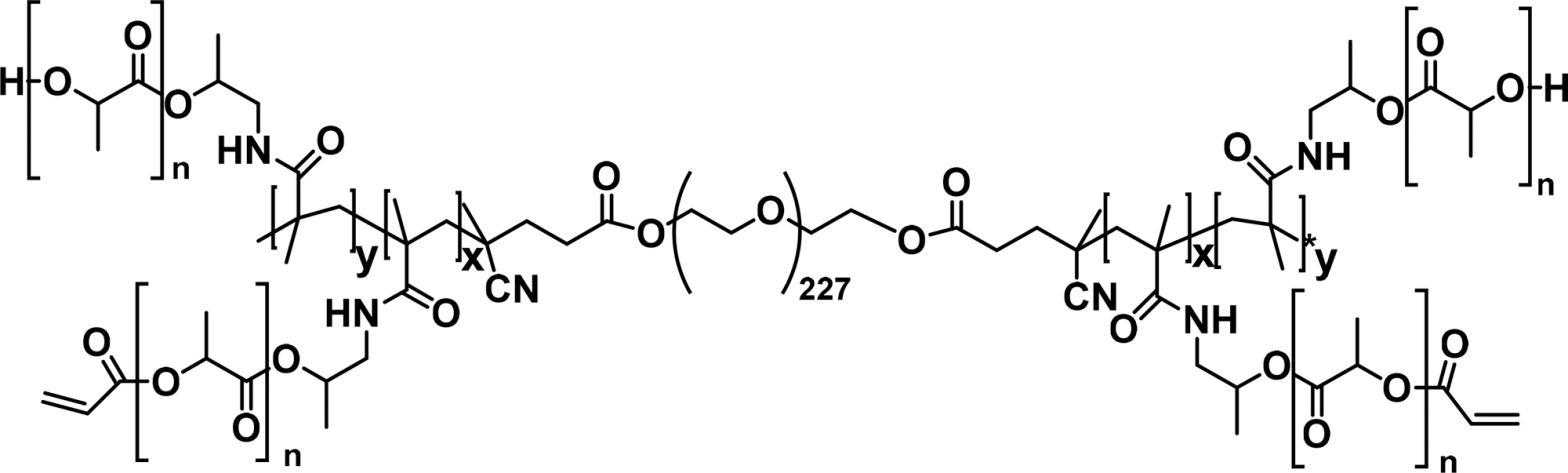
Chemical Structure of ABA-Triblock Copolymer Based on a Polyethylene Glycol (PEG_10000_) B-Block Flanked by Two Acrylated Poly(*N*-(2-hydroxypropyl) Methacrylamide Mono/Dilactate) (pHPMA-lac) A-Blocks Characteristics of the A-blocks: *M*_n_ was 14 kDa as determined by ^1^H-NMR (DMSO), molar ratio of mono/dilactate was 75:25 and the degree of acrylation was 15%. The average molecular weight (*M*_n_) of the ABA-triblock polymer (pHPta), determined by ^1^H-NMR (DMSO), was 38.8 kDa, and the cloud point was 15 °C.

All commercial chemicals were obtained from Sigma-Aldrich (Zwijndrecht, The Netherlands) and used as received unless indicated otherwise. Hyaluronic acid (HA, 31 kDa) was supplied by Lifecore Biomedical (Chaska, MN, USA) and thiolated as described previously,^31^ resulting in a degree of thiolation of 60% (HA-SH, structure shown in Scheme 1). The ABA-triblock copolymer consisting of a poly(ethylene glycol) (PEG, 10 kDa) midblock, flanked by thermosensitive blocks of partially acrylated *N*-(2-hydroxypropyl) methacrylamide-mono/dilactate (pHPMAmlac) (mono/dilactate molar ratio = 75:25) outer blocks was synthesized according to previously described methods (structure shown in Scheme 2).^31,33^ The degree of acrylation was 15%, referring to the percentage of OH side groups that was functionalized by acrylate moieties. The number-average molecular weight (*M*_n_) of the ABA-triblock polymer, determined by ^1^H NMR (DMSO), was 38.8 kDa. This polymer has an LCST of 15 °C, which means that upon heating an aqueous solution of this ABA-triblock polymer, abbreviated as pHPta, and HA-SH above the LCST to 37 °C, a gel is formed which is stabilized over time by Michael type addition reaction between thiol groups of HA-SH and acrylate groups of the thermosensitive pHPta. Once formed, the hydrogel degrades over time under physiological conditions due to the presence of hydrolytically sensitive ester bonds in the cross-links of the gel.^31^

The thermosensitive hydrogels were prepared by mixing the triblock copolymer in phosphate-buffered saline (PBS; 137 mM NaCl, 10 mM phosphate, 2.7 mM KCl, and a pH of 7.4) with a HA-SH stock solution in the same buffer at 37 °C. The concentrations were 15 wt % triblock and 4 wt % HA-SH for low concentration thermosensitive (LC Thermo) and 27 wt % triblock and 7 wt % HA-SH for high concentration thermosensitive (HC Thermo) hydrogels, corresponding to a molar ratio thiol/acrylate groups of 1:1. The polymers were dissolved and degassed as previously described.^31^ Solutions of both the triblock and HA-SH were chilled on ice before mixing in order to slow the reaction rate of the thiol groups and the acrylates, thus preventing cross-linking during handling.

To enable MRI-based measurement of release properties, gadolinium-labeled albumin (Galbumin, BioPAL Inc., Worcester, MA, USA) was loaded in the hydrogels. There are approximately 10 to 15 gadolinium atoms per albumin molecule and the final molecular weight is ∼74 kDa. The protein loaded hydrogels were prepared as follows: 2.4 mg (LC Thermo) or 4.2 mg (HC Thermo) of HA-SH were dissolved at 4 °C in 28.2 μL of PBS and 1.8 μL of Galbumin (25 mg/mL) to reach a volume of 30 μL (concentrations of HA-SH and Galbumin were 80 and 1.50 mg/mL, respectively). For the blank gels, HA-SH was dissolved in 30 μL of PBS. Next, 12.0 mg (LC Thermo) or 21.6 mg (HC Thermo) of thermosensitive triblock copolymer was dissolved in 40 μL PBS at 4 °C. Finally, 30 μL of the obtained thermosensitive triblock copolymer solution were transferred into a tube also containing the HA-SH with or without Galbumin solution at 4 °C (30 μL), thus bringing the final Galbumin concentration to 0.75 mg/mL (or 0 mg/mL for blank), and the final LC Thermo or HC Thermo concentration to 150 and 270 mg/mL, respectively. This solution was introduced in either tubes for MR imaging (200 μL test tubes containing 40 μL of hydrogel covered by 160 μL of PBS) or syringes for the *in vitro* and *in vivo* release studies, respectively. Gel formation was triggered by bringing the different formulations to 37 °C (*in vitro*) and incubation for 30 min or by injection into the brain.

### 1.2 Oscillatory Rheology Measurements

Bulk oscillatory rheology time-sweep measurements were conducted at 37 °C during 5 h on a stress-controlled AR-G2 rheometer equipped with a Peltier plate (TA Instruments, New Castle, Delaware, USA) with a 20 mm steel cone (1°) geometry equipped with a solvent trap, to obtain values for the storage and loss modules (*G*′ and *G*″, respectively). Rheological properties were examined at a frequency of 1 Hz at fixed strain of 1%. A solvent trap was used to prevent solvent evaporation. Experiments were repeated three times per sample and representative data are presented.

### 1.3 Hydrogel Injection in Rat Brain

All animal procedures were approved by the Ethical Committee on Animal Experiments of the University Medical Center Utrecht and Utrecht University, (DEC: 2012.I.11.116) and conducted in accordance with the guidelines set by the European Community Council Directives 86/609/EEC. Experiments are reported in compliance with the ARRIVE 2.0 guidelines (Animal Research: Reporting in Vivo Experiments).

Male Wistar rats (300–330 g) (Harlan, Horst, The Netherlands) were endotracheally intubated and mechanically ventilated with a mixture of 2% isoflurane in 70% air/30% O_2_ for anesthesia, and placed in a stereotaxic apparatus (Kopf Instrument, Tujunga, CA, USA). The skull was exposed between bregma and lambda. A burr hole was drilled using a dental drill during continuous irrigation with 0.9% saline at room temperature to prevent overheating of the underlying cortex.

Five microliters of each hydrogel formulation was stereotaxically injected over a 10 min period in the right striatum (stereotaxic coordinates from bregma (mm): AP, 0.3; ML, 3.0; DV, 4.0 from dura) using a 26-G Hamilton microsyringe (80330, Hamilton Company, Reno, NV). The following hydrogels were used: LC Thermo (blank: *n* = 2; Galbumin-loaded: *n* = 3) and HC Thermo (blank: *n* = 3; Galbumin-loaded: *n* = 3). In each case, the needle was left in place after injection for 7 min before being slowly withdrawn.

### 1.4 Behavioral Assessment

To assess possible adverse effects of intrastriatal hydrogel injection on animal behavior, rats were tested before stereotaxic injection and postinjection at days 2, 7, 14, and 21 using a modified sensorimotor performance score.^43^ This test was selected due to its sensitivity to detect gross neurologic and behavioral abnormalities. The test has seven limb-placing tasks, which assess forelimb and hindlimb responses to tactile and proprioceptive stimulation. Tactile stimulation was elicited by contacting the limb to a table surface, and proprioceptive stimulation was elicited by pulling down the limb (with limb position in space).^44^ Scores range from 0 (no deficits) to 9 (severe sensorimotor dysfunction). Behavioral testing was carried out by a person blinded to treatment.

### 1.5 MR Relaxation and Relaxivity Measurements

MRI measurements were performed on a 4.7 T/40 cm horizontal bore MR system (Agilent Technologies, Santa Clara, CA, USA) with a custom-built solenoid coil (3 cm diameter). *T*_1_ values of fresh samples of Galbumin with increasing concentrations (0.093, 0.19, 0.37, 0.74 mg/mL) in PBS or Galbumin in HC Thermo and LC Thermo hydrogels at 37 °C were measured directly after preparation, inside PCR tubes that were placed in a custom-made sample holder, using a Look-Locker-based inversion recovery protocol (8-shot gradient echo EPI; image repetition time (TR) 25 ms; number of images 100; flip angle 5°; inversion time 10 ms; echo time (TE) 4.2 ms; total TR 10000 ms; 100 images per 180° inversion pulse; 2 averages; 3 slices with 0.188 × 0.188 × 1 mm^3^ resolution).

Longitudinal relaxivity *r*_1_ (mM^–1^ s^–1^) of the contrast agent in PBS, LC thermo, and HC thermo was calculated from the measured *T*_1_ values based on the following equation:
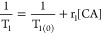
1where *T*_1(0)_ is the *T*_1_ value (s) without contrast agent and [CA] is the concentration of the contrast agent (mM).^45^

### 1.6 MRI of *In Vitro* Protein Release From Hydrogels

PCR tubes were filled with 40 μL of hydrogel, equilibrated for 30 min at 37 °C, and subsequently covered with 160 μL of PBS at 37 °C. Next, samples were placed in a custom-made sample holder and transferred into the MR scanner for 24 h monitoring. The sample temperature during MRI and in between MRI sessions was maintained at 37 °C up to 27 days after preparation, and PBS supernatant was refreshed 24 h before each subsequent MRI measurement. The MRI measurements of nonlabeled hydrogel (*n* = 3) and Galbumin-loaded hydrogel (*n* = 3) samples from the two hydrogel formulations (LC Thermo and HC Thermo) were performed at 4.7 T in the same solenoid coil used for relaxivity measurements. *T*_1_ maps were acquired using the Look-Locker-based inversion recovery protocol that was identical to the protocol used for the in vitro relaxivity measurements (Look-Locker 8-shot gradient echo EPI; image repetition time (TR) 25 ms; number of images 100; flip angle 5°; inversion time 10 ms; echo time (TE) 4.2 ms; total TR 10000 ms; 100 images per 180° inversion pulse; 2 averages; 3 slices with 0.188 × 0.188 × 1 mm^3^ resolution). Serial T_1_ maps were continuously acquired from 1.5 to 24 h after preparation to measure Galbumin release. In addition, gradient echo 3D (GE3D) MRI (TR/TE 10/2.57 ms; pulse angle 20°; FOV 40 × 40 × 40 mm^3^; data matrix 128 × 128 × 256; voxel resolution 0.313 × 0.313 × 0.156 mm^3^) and spin echo (SE) MRI (TR/TE 500/15.54 ms; 2 averages; 3 slices; voxel resolution 0.188 × 0.18 × 1 mm^3^) data were collected for assessment of gel volume and delineation of regions-of-interest (ROIs), respectively. Additional T_1_ maps, GE3D and SE images were acquired at 2, 4, 6, 9, 12, 21, and 27 days after preparation, using identical imaging protocols.

### 1.7 MRI of *In Vivo* Protein Release From Intracerebrally Injected Hydrogel Formulations

*In vivo* MRI measurements were performed on a 4.7 T/40 cm horizontal bore MR system (Agilent Technologies, Santa Clara, CA, USA) with a custom-built (2.5 cm diameter) surface coil for signal reception, and a Helmholtz volume coil for signal transmission. Rats were secured in a MR-compatible cradle using ear bars and a bite bar, and mechanically ventilated with a mixture of 2% isoflurane in 70% air/30% O_2_ for anesthesia. Blood oxygenation, expired CO_2_, and heart rate were continuously measured throughout imaging and remained within physiological ranges. The core temperature was maintained at 37.0 ± 0.5 °C. Serial MRI was performed directly after, and 2, 7, 14, and 21 days after stereotaxic hydrogel injection. GE3D MR images, similar to those described for the *in vitro* release measurement, were collected to localize the injected hydrogel. T_1_ maps were acquired from a Look-Locker-based inversion recovery protocol with EPI read-out (8 shots; image TR 25 ms; flip angle 5°; inversion time 10 ms; TE 8.75 ms; total TR 6000 ms; 28 images per 180° inversion pulse; 24 averages 5 slices with 0.15 × 0.15 × 1 mm^3^ resolution).

### 1.8 Image Processing and Analysis

*T*_1_ maps were obtained by voxel-wise three-parameter fitting of the Look-Locker-based inversion recovery magnitude images in Matlab (MathWorks, Natick, MA, USA), using the equation as described by Deichmann and Haasse.^67^
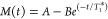
2where *T*_1_* is the effective relaxation time, *A* = *M*_0_* and *B* = *M*_0_ + *M*_0_*. True *T*_1_ values were calculated from the fitted parameters using the following equation:
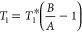
3To assess hydrogel swelling or shrinkage under *in vitro* and *in vivo* conditions, hydrogel volumes were automatically segmented using a region-growing algorithm.^46^ A seed voxel was selected in the center of the gel on GE3D images (*in vitro* study) or T_1_ maps (*in vivo* study), after which neighboring voxels with similar intensity or *T*_1_ values were automatically added.^47^

**Figure 1:**
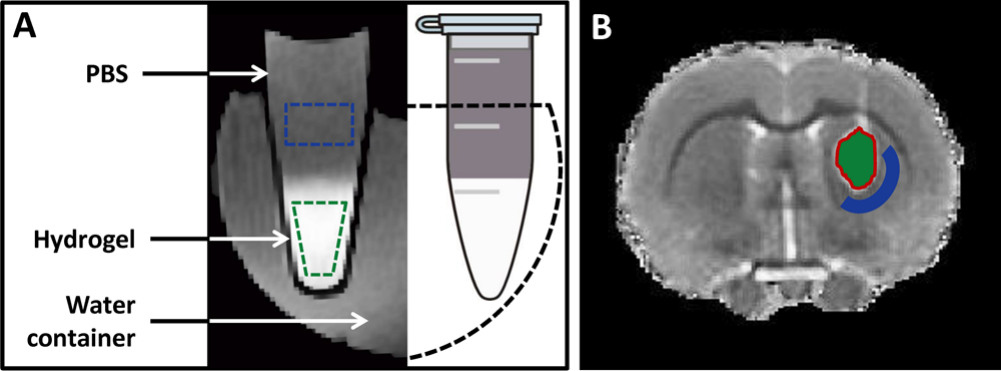
Regions-of-interest on *in vitro* and *in vivo* MR images of hydrogels: (A) Illustration of *in vitro* MRI setup for measurement of the release of Galbumin from hydrogels (identified at the bottom of the tube by high signal intensity on GE3D image) into the supernatant. Regions-of-interest (ROIs) are depicted in the hydrogel (green) and the PBS supernatant (blue). (B) *T*_1_ map of a coronal rat brain slice after stereotaxic injection of nonlabeled hydrogel in the right striatum. The corresponding ROIs for further image analysis are overlaid on the image. Green color represents implanted hydrogel; blue is surrounding peri-injection tissue. The red line indicates the 2-voxel wide rim area that was excluded from analyses.

For the *in vitro* MRI study, two ROIs were manually outlined on the SE images for each sample, i.e. one inside the hydrogel and one in the PBS supernatant (Figure 1A). For the *in vivo* MRI study, two ipsilateral ROIs were manually outlined on the multislice *R*_1_ (1/T_1_) maps, i.e. one inside the hydrogel at the injection site (Figure 1B) and one in an adjacent tissue area in the striatum. The border of the ROI inside the hydrogel was kept at 2 voxels distance from the hydrogel’s rim area. The adjacent tissue ROI was drawn at 10 voxels from the outer rim of the hydrogel. These ROIs were mirrored to the contralateral hemisphere. For each time point, mean *R*_1_ values for the ROIs were converted to Galbumin concentration, based on the differences in *R*_1_ values between the nonlabeled and Galbumin-labeled samples and the corresponding relaxivities according to the following formula:
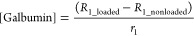
4where *R*_1_ is the relaxation rate (1/*T*_1_) and *r*_1_ is the longitudinal relaxivity in PBS, LC Thermo, or HC Thermo.

We calculated apparent (measured) and corrected Galbumin release from the hydrogels by taking into account the volume changes *in vitro* (at 37 °C). Briefly, Galbumin release from the hydrogels (% of initial dose in the gel at the first MRI time point) was calculated from the hydrogel, using the following equation:
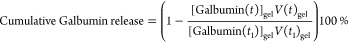
5where *V*(*t*_1_)_gel_ and *V*(*t*)_gel_ are the gel volumes at the first MRI time point *t*_1_ and at subsequent time points *t*, respectively.

The *in vitro* release rate constants from the gels were assessed by linear regression analysis of the cumulative Galbumin release versus the square root of time.^48,49^ In order to further establish the nature of the release mechanism, the mesh size of the hydrogels was calculated using the following equation:
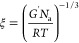
6where *G*′ is the storage modulus, *N*_a_ is the Avogadro’s number, *R* is the gas constant, and *T* is the temperature in K.^42^

An estimate of the diffusion coefficients of Galbumin in the two thermosensitive hydrogels were calculated using a modified form of the Fick’s law for short release times from a slab of gel using the formula:^48,49^
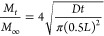
7where *M*_*t*_/*M*_∞_ is the cumulative % released at time *t* (in seconds), *D* is the diffusion coefficient of Galbumin within the system, and *L* represents the hydrogel slab thickness at the bottom of the PCR test tube (*L* = 5.5 mm).

### 1.9 Statistical Analysis

The results are presented as the means ± the standard deviation. A one-way repeated measures analysis of variance (ANOVA) followed by Bonferroni’s post-testing was done to identify significant differences between groups for each time point. Statistical analysis of *T*_1_ of surrounding ipsilateral and homologous contralateral brain tissue at different time points after unilateral implantation of hydrogels was performed using two-way repeated measures ANOVA with Bonferroni’s post-testing. *P* values of less than 0.05 were considered statistically significant.

## Results and Discussion

### 2.1 Gel Formation and Rheological Properties

**Figure 2:**
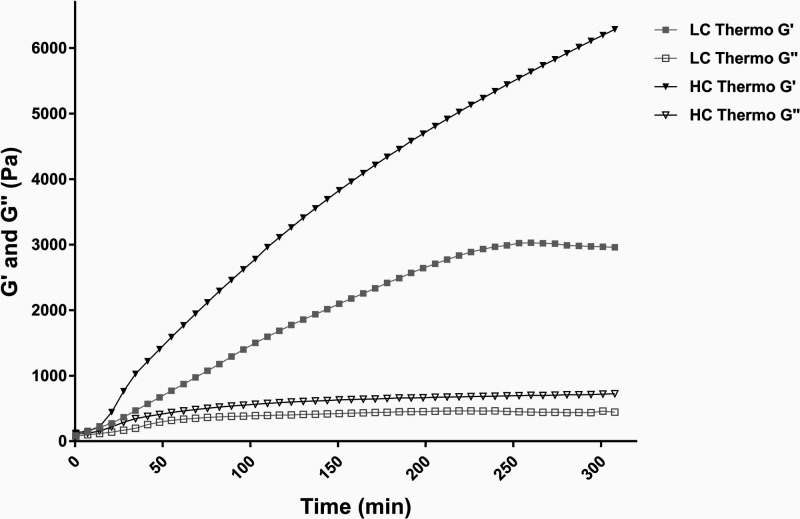
Rheological properties of the hydrogels. Storage modulus (*G*′) and loss modulus (*G*″) of low concentration (LC Thermo) and high concentration (HC Thermo) thermosensitive hydrogels in time measured in situ for 5 h at 37 °C. At time = 0, the liquid pregel samples were inserted in the rheometer.

The polymer gelation behavior of the two different Thermo gels was quantitatively assessed by rheological measurements. Figure 2 shows the storage modulus (*G*′) and loss modulus (*G*″) of the gels as a function of time, where time = 0 min is the moment of inserting liquid pregel in the rheometer at 37 °C. The storage modulus (*G*′) increased steeply over time for the HC Thermo gel and reached a value of 6320 Pa at the end of the experiment (after 310 min). The loss modulus (*G*″) reached a value of 721 Pa. The LC Thermo gel on the other hand, had a more gradual increase of *G*′ to about 3000 Pa (obtained after 230 min), which remained relatively constant until end point. The loss modulus (*G*″) reached a value of 461 Pa.

### 2.2 Longitudinal Relaxivity

The longitudinal relaxivity of an MR contrast agent represents its potency to generate contrast in *T*_1_-weighted MR images. The longitudinal relaxivities (*r*_1_) of Galbumin in PBS, LC Thermo, and HC Thermo hydrogels, determined from the dependence of the measured *R*_1_ relaxation rate on the contrast agent concentration at 37 °C, were 95.5 ± 5.6, 93.2 ± 0.4, and 63.2 ± 3.9 mM^–1^ s^–1^, respectively. Based on the relaxivity measurements, incorporation of 0.75 mg/mL Galbumin in the two hydrogels was selected for an optimal signal-to-noise ratio.

### 2.3 MRI Volumetry

The swelling of implanted hydrogels may have an impact on their mechanical properties. For CNS delivery, *in vivo* swelling is a particularly critical confounder that could exacerbate brain injury. Volume changes of hydrogels are structurally predetermined from the nature of the composing polymer chains and their cross-link density, and can also be strongly affected by *in vivo* environmental conditions, external pH, ionic strength, temperature stimuli, etc.^38^ Thus, understanding the swelling behavior of hydrogels *in vitro* as well as *in vivo* is important from both theoretical and practical points of view for drug delivery applications.

**Figure 3:**
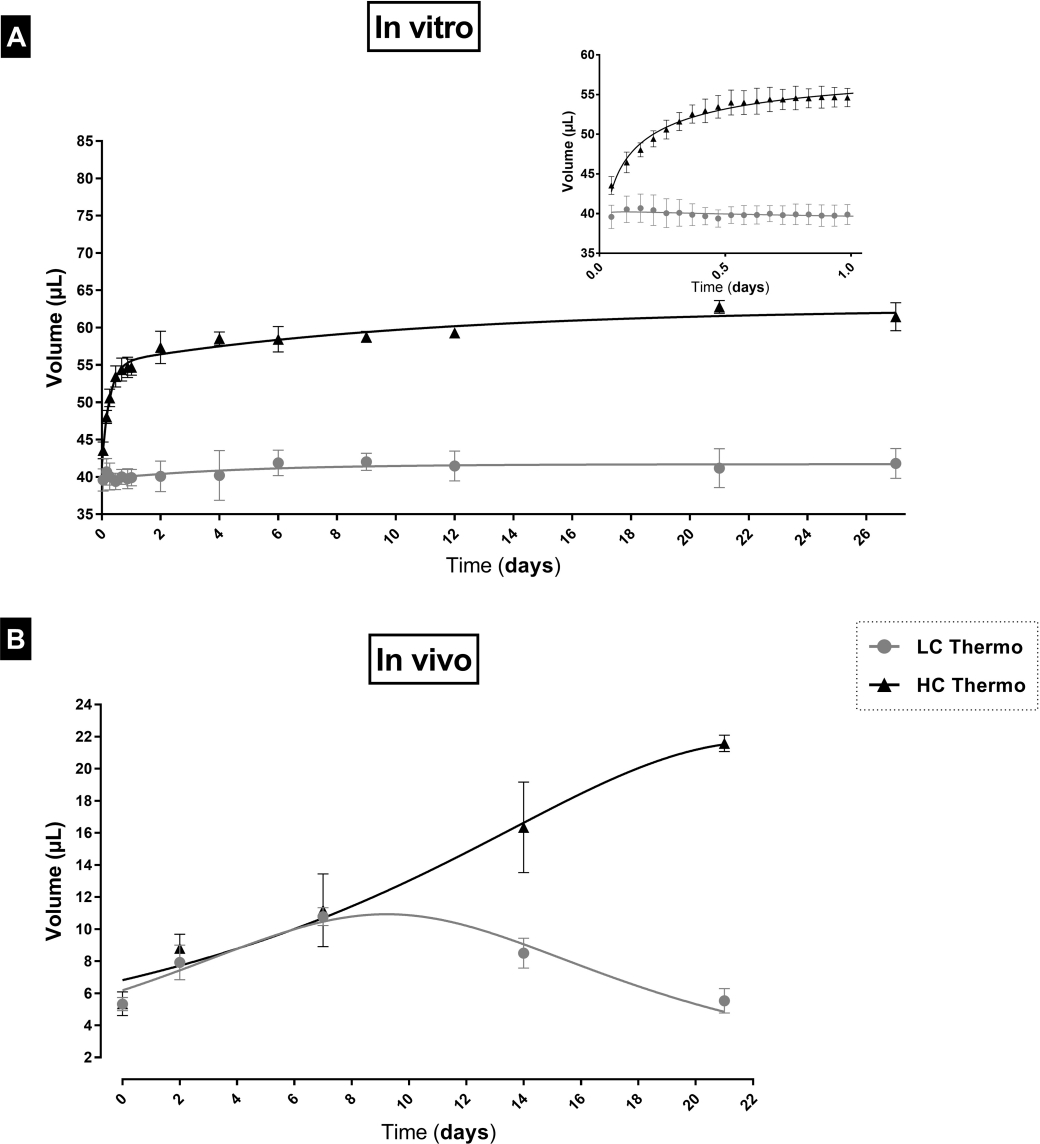
MRI volumetry of LC Thermo and HC Thermo gels: (A) *in vitro*, from day 0 to day 27, and (B) *in vivo*, from day 0 to day 21. *In vitro* measurements were started 75 min after the initiation of the gelation process. The first *in vivo* MRI data was acquired approximately 60 min after the stereotaxic gel injection. Volumetric analysis was performed on GE3D images and *T*_1_ maps for the *in vitro* and *in vivo* MRI measurements, respectively. Data are shown as mean ± SD (*n* = 3).

*In vitro*, the gels were identified as bulk materials deposited on the bottom of the tube with relatively high signal intensity on GE3D images, and covered with PBS with lower signal intensity (see Figure 1A). The high signal intensity of the gels resulted from the Galbumin-induced reduction in *T*_1_ relaxation time. The initial volume of each sample in liquid state was 40 μL. The measured volumes after completion of the first MRI session (*V*_0_), approximately 70 min after initiation of the gelation process, were 39.6 ± 1.5 and 43.5 ± 1.1 μL for the LC and HC Thermo gels, respectively (Figure 3A). The HC Thermo gel gradually increased in volume and reached a value of 54.7 ± 1.1 μL after 24 h. On the other hand, the LC Thermo gel was dimensionally stable with a volume of 40.4 ± 1.1 μL during the same time frame. At the final MRI time point, i.e. 27 days after preparation, the volumes of the LC Thermo and HC Thermo hydrogels were 41.8 ± 2.0 μL and 61.5 ± 1.9 μL, respectively (Figure 3A).

*In vivo*, at 1 h after stereotaxically injecting 5 μL of hydrogel, the volumes of the LC and HC Thermo gels were 5.3 ± 0.4 and 5.3 ± 0.7 μL, respectively (Figure 3B). In time, the LC Thermo gel increased in volume up to 10.8 ± 0.6 μL 7 days postadministration, followed by a volume decrease to 5.5 ± 0.8 μL by the end of the experiment after 3 weeks, likely due to degradation. The HC Thermo gel volume increased up to day 21 to 21.6 ± 0.5 μL. Thus, our results demonstrate a substantial discrepancy between *in vivo* and *in vitro* volume changes. This can probably be explained by differences in the volume (5 μL *in vivo* versus 40 μL *in vitro*) and surface area of the gels, as well as by differences in the properties of the environment. *In vitro*, the hydrogel, which is confined to the bottom of the test tube, had access to the PBS almost exclusively from the top, leaving this the only possible direction to swell. On the other hand, when injected in the brain tissue, it could theoretically expand in every direction with less constraint.

### 2.4 Assessment of *In Vitro* Protein Release Using MRI

The release of Galbumin from LC and HC Thermo gels in PBS at 37 °C was evaluated with serial MRI. The first set of GE3D images and corresponding *T*_1_ maps were obtained approximately 1 h after initiation of the gelation process.

**Figure 4:**
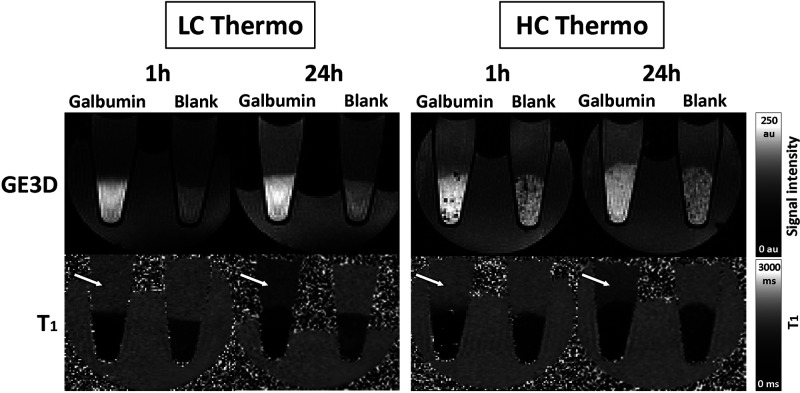
MRI of protein release from *in vitro* hydrogel samples: GE3D images (top) and *T*_1_ maps (bottom) of PCR tubes filled with 40 μL of Galbumin-loaded and nonloaded (blank) LC Thermo and HC Thermo gels, covered with 160 μL of PBS. The images were acquired 1 (left) and 24 (right) h after gelation. The signal intensity of the supernatant increased in GE3D images and *T*_1_ values in supernatant decreased (*T*_1_ maps) with time as a result of Galbumin release (white arrows).

Figure 4 shows representative GE3D images and *T*_1_ maps of the tubes at approximately 1 and 24 h after hydrogel preparation. Nonloaded (blank) hydrogels displayed a slight increase in *T*_1_ over time, most probably due to leakage of hydrogel building blocks not incorporated in the formed network into the supernatant. In contrast, Galbumin-loaded hydrogels showed a more substantial increase in *T*_1_ as a function of time, which is due to release of Galbumin from the hydrogel.

**Figure 5:**
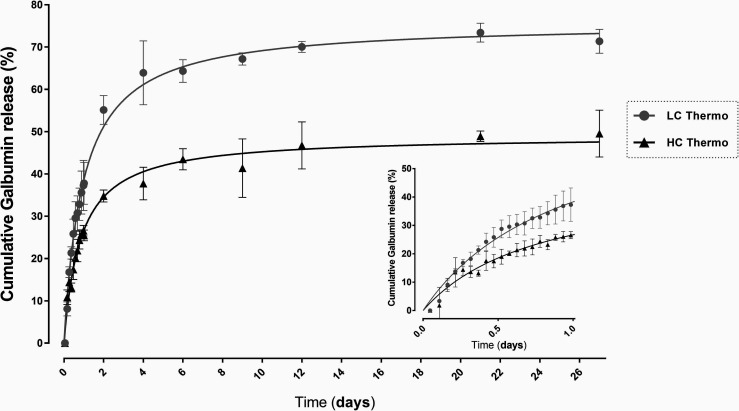
*In vitro* release of Galbumin from the LC and HC hydrogels: *in vitro* cumulative release (%) of Galbumin from the LC Thermo and HC Thermo gels at 37 °C over 27 days after gelation. Each point represents the mean value ± SD (*n* = 3).

The released Galbumin from the gels in % of the initial loading was calculated from the *T*_1_ values using formulas 4 and 5 (Materials and Methods, section 1.8). Figure 5 shows the release profile of the gels, where LC Thermo gel had a faster release rate.

The first 24 h showed a rapid release of Galbumin from both hydrogels, where 38 ± 6% of the Galbumin content in the LC Thermo gel and 25 ± 1% of that in HC Thermo gel was discharged. PBS in the tubes was refreshed thereafter 24 h before each scan in order to prevent any effect of increasing Galbumin concentration in the medium on the free diffusion from the gel. During the following days, the concentration of Galbumin in the thermosensitive hydrogels progressively decreased, corresponding with 64 ± 3% and 43 ± 3% release of their initial load from the LC and HC Thermo gel, respectively at day 6. After that, the release seemed to slow down, and at the end point, day 27, the Galbumin released was 71 ± 2% for LC Thermo and 50 ± 6.0% for HC Thermo gels. The residual amount of Galbumin at day 27 may probably be explained by the formation of chemical bonds between Galbumin and the hydrogel. Free thiol groups (HA-SH) and acrylates react spontaneously. However, in the presence of a protein, such as Galbumin, the acrylates may also react partially (with less affinity) with the amino groups of the protein. The higher the polymer concentration, the more free acrylates available to react with Galbumin, which may explain the differences in residual Galbumin amounts between LC and HC Thermo gels.

**Figure 6:**
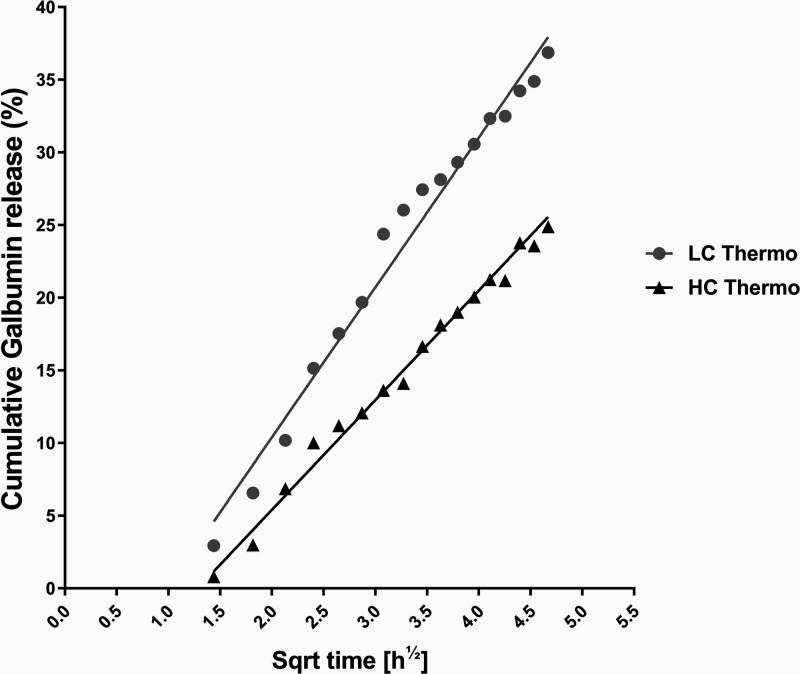
*In vitro*Galbumin release from the hydrogels at 37 °C: Galbumin concentration of LC Thermo and HC Thermo as a function of the square root of time up to 24 h after gelation. Each point represents the mean of three samples. The lines represent the linear fit of the data.

The cumulative fraction of Galbumin released from the gel into the supernatant was calculated with eq 5, and scaled linearly with the square root of time (*R*^2^ > 0.98) over the first 24 h after gelation (Figure 6). Linear correlation with the square root of time reflects first order kinetics, signifying that the release is governed predominantly by diffusion.^49,50^ In the first 24 h, release rate constants of Galbumin from LC Thermo and HC Thermo were 10.3 ± 0.3 and 7.5 ± 0.2% per hour^1/2^, respectively. Diffusion of Galbumin was significantly faster from the LC Thermo gel as compared to the HC Thermo gel (*F*(DFn, DFd) = *F*(1, 72) = 1105, *p* < 0.0001).

The diffusion based release mechanism was verified by calculating the mesh size of the hydrogel and comparing it with the size of albumin, using eq 6. Based on the approximate *G*′ value of 3.0 and 6.3 kPa for LC Thermo and HC Thermo, respectively, we estimated a mesh size of 11.3 and 8.8 nm. The mean hydrodynamic diameter of albumin is 7.2 nm,^51^ which is marginally smaller than the mesh size of both hydrogels, supporting the diffusion based release kinetics of the protein. Based on a simplified method calculation, the diffusion coefficient of Galbumin in the hydrogel was determined to be a factor 10–15 slower compared to the diffusion in water (see Supporting Information).

### 2.5 *in Vivo* Protein Release Evaluated by MRI

**Figure 7:**
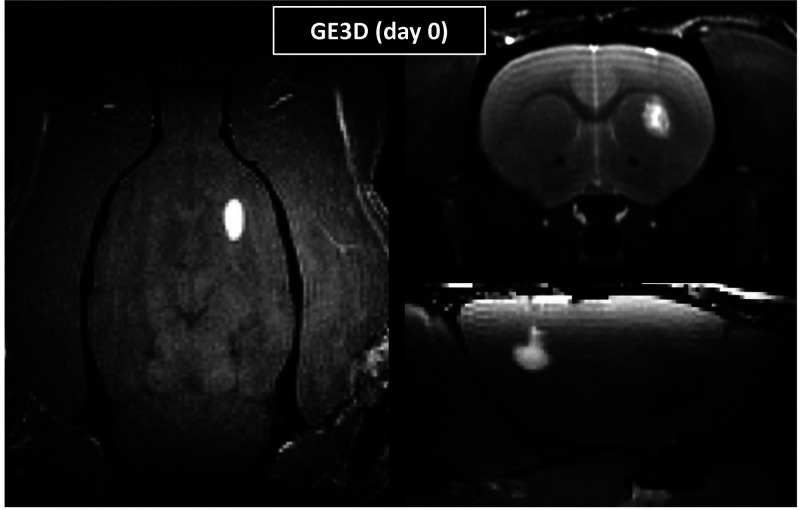
*In vivo* MRI of hydrogels after injection in rat brain. Representative GE3D images of axial (left), coronal (top right), and sagittal (bottom right) rat brain sections directly after stereotaxic Galbumin-loaded gel (LC Thermo) injection into the right striatum.

**Figure 8:**
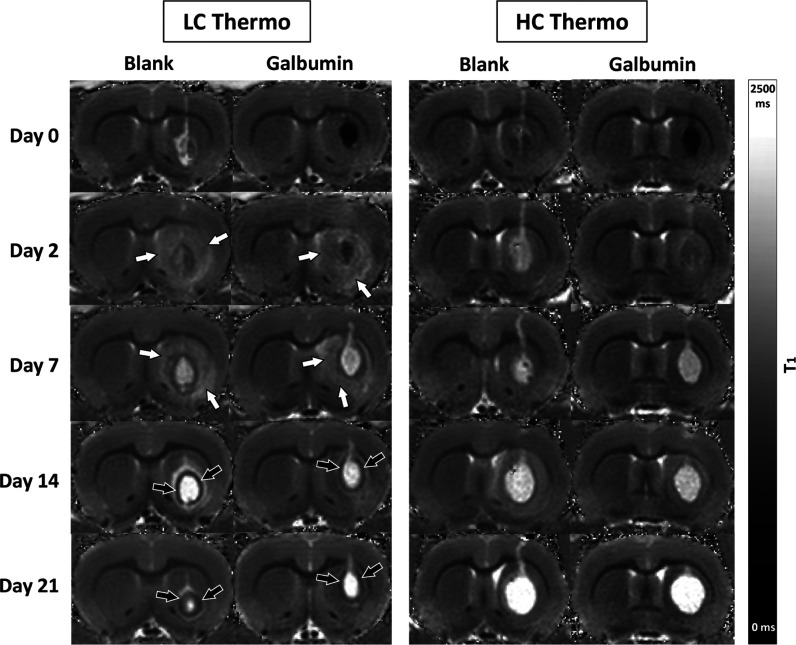
Serial *T*_1_ maps of rat brain after injection of different blank or Galbumin-loaded hydrogels. *T*_1_ maps of a coronal rat brain slice at different time points after stereotaxic injection of nonloaded (blank) or Galbumin-loaded hydrogel in the right striatum. Directly after injection (day 0), the Galbumin-loaded hydrogel appears as an area with relatively low *T*_1_ values due to significant Galbumin-induced *T*_1_ shortening. Hydrogel volume remained relatively stable (LC Thermo), or increased (HC Thermo), up to day 21 after injection. *T*_1_ normalization in Galbumin-loaded thermosensitive gels during the first week after injection pointed toward release of Galbumin. A significant prolongation of *T*_1_ was observed in tissue surrounding the injection area of Galbumin-loaded or blank LC Thermo gel on day 2 and 7 after implantation (white arrows). Additionally, a prominent rim with relatively low *T*_1_ values enveloping the LC Thermo gels injection site formed around day 7 and remained present up to day 21 (black arrows).

The hydrogels injected in the center of the striatum were clearly detectable on *in vivo* rat brain GE3D images (Figure 7). Serial MRI over the 21 days observation period revealed clear changes in *T*_1_ values at the hydrogel injection site (Figure 8). Significant *T*_1_ shortening in the paramagnetic Galbumin-loaded gels resulted in a hypointense appearance on *T*_1_ maps. The blank LC and HC Thermo gels showed a gradual *T*_1_ prolongation toward the final measurement on day 21, when the injected gels appeared as distinctive bright volumes on *T*_1_ maps. Besides swelling, degradation of the gel matrix likely contributes to the *T*_1_ prolongation. Based on previous data, we do not expect degradation of the hydrogels within the time window of the *in vitro* (27 days) or the *in vivo* experiments (21 days).^31^ In the Galbumin-loaded thermosensitive gels, *T*_1_ normalized during the first week, pointing toward Galbumin release, followed by further *T*_1_ prolongation up to day 21.

Biomaterials science is striving to design hydrogels that are biocompatible with the implanted tissue environment. Often, injected materials induce a foreign body reaction, and the inflammatory response varies depending on the material.^53^ Some natural polymers, such as hyaluronic acid (HA) in thermosensitive hydrogels, have intrinsic anti-inflammatory properties, which may negate inflammation at the site of delivery.^54^ Interestingly, after injection of Galbumin-loaded or blank LC Thermo gel, a significant increase of *T*_1_ was observed in tissue surrounding the injection area on day 2 and 7 after implantation, which was less obvious or absent after implantation of the HC Thermal gel (Figure 8, white arrows, and Figure 10). This *T*_1_ prolongation in adjacent tissue may have been caused by elevated fluid accumulation. Additionally, a marked rim with relatively short *T*_1_, enveloping the LC Thermo gels injection site, formed around day 7 and remained present up to the final MRI measurement (Figure 8, black arrows). This effect, which was not obvious after injection of the HC Thermo gel, may reflect gliosis.^55^

**Figure 9:**
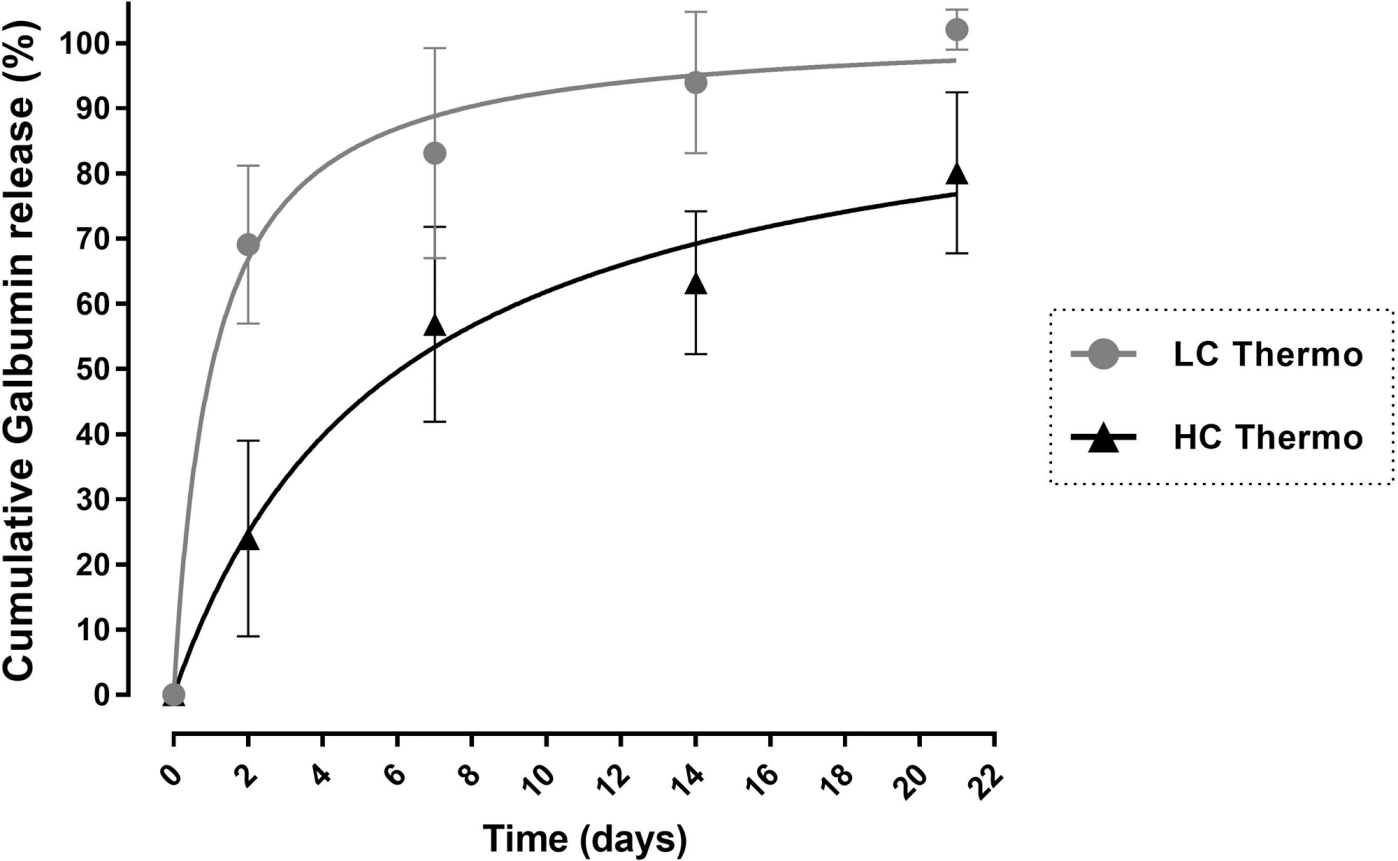
*In vivo* Galbumin release from intracerebrally injected hydrogels. *In vivo* cumulative Galbumin release from LC Thermo and HC Thermo gels as a function of time after intracerebral injection in a rat brain. Data are shown as mean ± SD (LC Thermo, *n* = 3; HC Thermo, *n* = 3).

The success of a biocompatible drug delivery system depends on its ability to deliver pharmacologically active compounds to the treatment area at specific rates, throughout the entire therapy regimen.^12^*In vivo* Galbumin release from the hydrogels was quantified from the *T*_1_ measurements, similar to the *in vitro* MRI experiments. We corrected for volume changes in order to standardize the release kinetics between the two hydrogels, in the same way as for the *in vitro* experiment (eq 5) and present the results in a Galbumin release graph (Figure 9). Within 2 days after intracerebral injection, the LC Thermo and HC Thermo gels lost 69 ± 12% and 24 ± 15% of their initial protein load (after volume correction), respectively. In the subsequent weeks, the gradual Galbumin release persisted, resulting in residual levels (after volume correction) of approximately 0% and 20% at 21 days postinjection for LC Thermo and HC Thermo gels, respectively.

Theoretically, a relatively high polymer weight fraction is expected to yield a gel with slower degradation kinetics and prolonged release of the loaded protein compared to a gel with a lower polymer weight fraction.^56,57^ In line with this, the HC Thermo gel, having a higher polymer weight fraction, demonstrated slower Galbumin release both *in vitro* (Figure 5) and *in vivo* (Figure 9) compared to the LC Thermo gel. Both gels demonstrated first order release kinetics *in vitro*, meaning that the size of the protein is smaller than the mesh size of the hydrogel matrices. Moreover, for both gels, Galbumin release reached higher values *in vivo* after intracerebral implantation of the gel, compared to *in vitro*. Until the end of the first week after intracerebral injection, LC Thermo and HC Thermo gels had already released 83 ± 16% and 57 ± 15% of their protein loading into the brain, respectively. This may have been caused by the specificity of the tissue environment^58^ and the potential enzymatic degradation of the polymer matrix *in vivo*,^59,60^ increasing the release rate compared to *in vitro* conditions. In particular, the action of hyaluronidases in the brain may be responsible for cleaving the HA component of the thermosensitive hydrogel network.^61^ Indeed, *in vivo* and *in vitro* correlation of hydrogel controlled release systems has been hardly investigated, and may be affected by the environment at the site of administration. Assessment of *in vitro* release kinetics describes a hydrogel formed under idealized conditions of pure buffer solutions, in the absence of tissue, CSF or blood factors that are present *in vivo*. *In vivo* conditions that affect drug release can be independent of the delivery system, such as, drug diffusion barriers (fluid viscosity, connective tissue), drug partitioning at the site (possible uptake into fatty tissue), the fluid volume present at the site and specific tissue dynamics.^62^ Delivery system dependent factors, on the other hand are specific to a particular delivery system and range from enzymatic degradation, protein adsorption, phagocytosis, as well as any possible inflammatory reaction to the delivery system.^62^ With the thermosensitive hydrogel in this study, the smaller volume used *in vivo* (5 μL) compared to *in vitro* (40 μL), resulting in a larger surface/volume ratio, may have influenced the swelling behavior and release kinetics. Also, potential dilution may have occurred during injection in the brain while the gel was formed, which is absent during *in vitro* hydrogel formation, since no excess fluid was present and release medium was added at a later time point.

**Figure 10:**
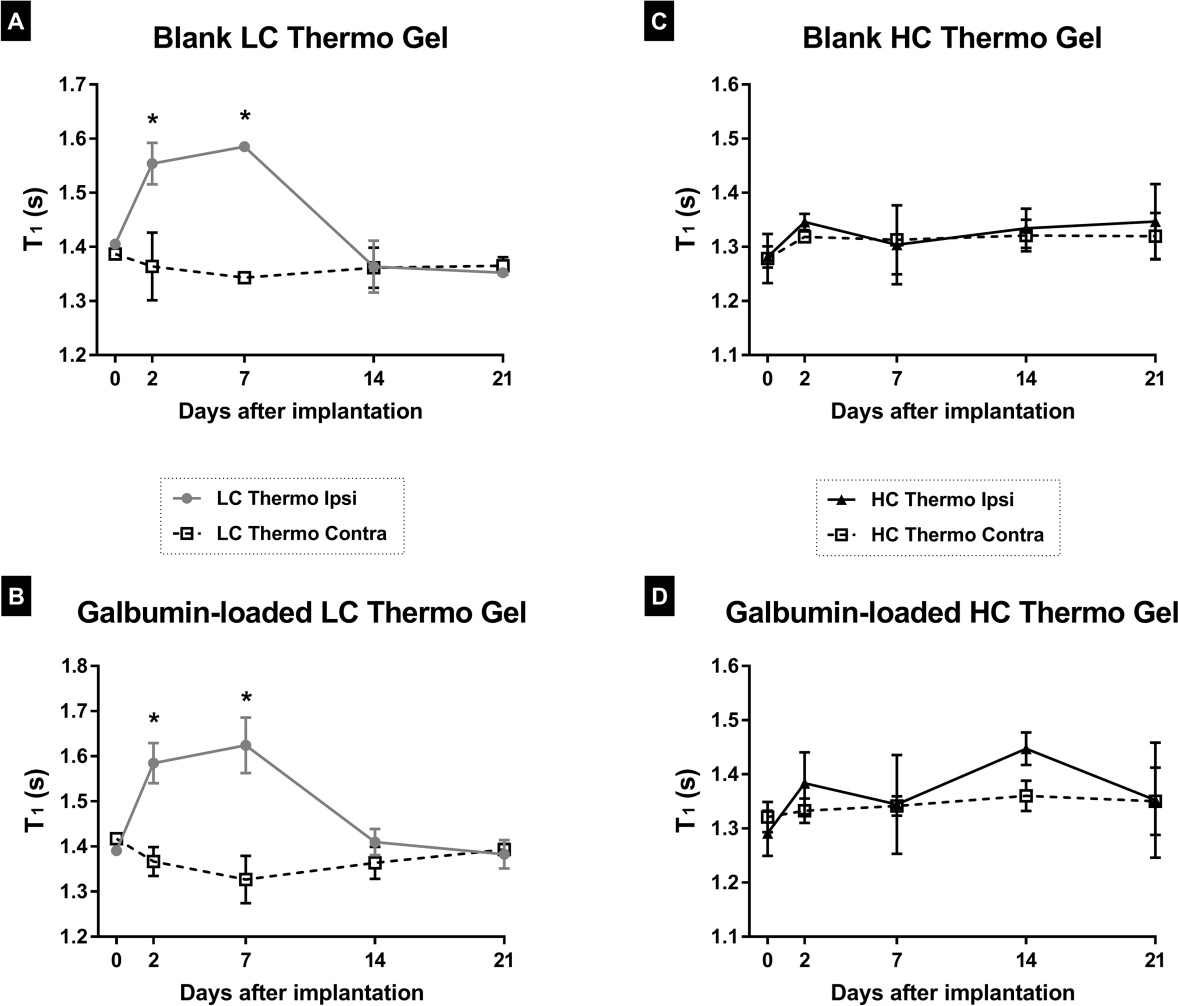
*T*_1_ of surrounding ipsilateral and homologous contralateral brain tissue at different time points after unilateral implantation of the hydrogels. *P < 0.05 vs day 0.

The thermosensitive gels used in the present study were first described by Censi et al., and were suggested as a slow release systems suitable for *in vivo* applications.^31^ Yet, the behavior of these hydrogels in the brain have not been previously addressed. In the present study, the high concentration (HC) Thermo gel had a large swelling ratio, particularly after implantation in the brain. Biomaterials with enhanced capacity to absorb water from the surrounding environment are prone to have increased contact with the ECM, however an uncontrolled swelling profile may affect the structural stability of the material, causing compressive pressures on the surrounding tissue.^63^ This, on the other hand may evoke a local tissue response and cerebral edema formation that may be detected with *T*_1_ MRI. Changes in *T*_1_ values outside the area of gel implantation were not detected for the HC Thermo gel. However, we did find an increase in *T*_1_ values after injection of the LC Thermo gel, both in the absence and presence of Galbumin loading (Figure 10). Because LC Thermo gel degrades and releases its contents faster than the HC Thermo gel, we speculate that *T*_1_ prolongation in adjacent tissue was at least partly due to elevated fluid accumulation potentially as a result of discharged degradation products. These degradation products diffuse into the surrounding tissue, thereby increasing the water content in the extracellular space via osmosis. Additionally, it may be possible that the injection procedure and/or the faster degradation of the LC Thermo gel evokes an inflammatory response prompting glial activation around the gel.^64^ Thus, the rim with relatively short *T*_1_ around the hydrogel may be similar to MRI-based observations of a fibrous pseudocapsule as a result of scar tissue formation in the case of tissue gel implants, such as dermal fillers and mammoplasty.^65,66^ In the chronic phase of inflammation, gliosis may result in isolation of the delivery system and a consequent reduction in the fluid volume, which in turn will alter the release kinetics.^62^ The rim-like *T*_1_ shortening around the gel cannot be explained by a peripheral retention of Galbumin, as the same feature was observed around the blank LC Thermo gel implants (Figure 8).

The observed *in vivo* volume increase for both thermosensitive gels, accompanied by signs of fluid accumulation in surrounding tissue of implanted LC Thermo gels at day 2 and 7, did not result in significant behavioral changes in the sensorimotor performance score (SPS). SPS values were 0 ± 0 (i.e., no deficits) for all experimental groups at all time points (data not shown).

## Conclusion

While many hydrogel-based drug delivery systems have been developed for localized treatment, the scarcity of noninvasive imaging techniques to get insight into the relationship between *in vitro* and *in vivo* release behavior remains a limiting factor. The MRI methodology presented in this study provides a unique approach to noninvasively monitor controlled protein release from a locally injected hydrogel formulation into the brain of living animals. This strategy allowed the longitudinal assessment in individual animals, therefore substantially decreasing the number of experimental animals required to obtain high-quality data. After injection into the rat brain, the release kinetics of a gadolinium-labeled model protein (Galbumin) from two thermosensitive hydrogels of the same chemical composition but different initial water content could be determined by changes in *T*_1_ up to 3 weeks after implantation. The evaluation of discrepancies between *in vitro* and *in vivo* release profiles and volume changes suggests that the local microenvironment may have a significant impact on hydrogel behavior. Moreover, within the same imaging session, characterization of hydrogel behavior can be done in conjunction with an estimation of the effects on the surrounding tissue using structural and functional imaging protocols.

Our MRI approach enables noninvasive release profile measurement of not only albumin but also other MR contrast agent-associated proteins from injected hydrogel matrices. This could aid in the development of innovative tailor-made carrier systems for prolonged drug release in the injured brain.

## Supporting Information Available

Supplementary Figure 1, Galbumin release from in vitro hydrogel samples at 37 °C over the first 24 h after gelation; Supplementary Figure 2, *T*_1_ maps of a coronal brain slice for each rat included in the study, acquired directly after stereotaxic injection of 5 μL thermosensitive gel in healthy rat brain; diffusion coefficient estimation using mathematical models for diffusion from a slab of gel in a reservoir system (PDF)
