# Supplementary material for: Thermosensitive Biodegradable Hydrogels for Local and Controlled Cerebral Delivery of Proteins: MRI-Based Monitoring of In Vitro and In Vivo Protein Release
Source: ACS Biomater Sci Eng. 2023 Jan 22;9(2):760–72. doi: 10.1021/acsbiomaterials.2c01224 (PMC9930091; doi:10.1021/acsbiomaterials.2c01224)
Supplement: Supplementary file 1 — ab2c01224_si_001.pdf [file ab2c01224_si_001.pdf]

Thermosensitive biodegradable hydrogels for local and controlled cerebral delivery of proteins: MRI-based monitoring of *in vitro* and *in vivo* protein release

Pavel Yanev[a,c], Geralda A.F. van Tilborg[a*], Kristel W.M. Boere[b], Ann M. Stowe[c], Annette van der Toorn[a], Max A. Viergever[a], Wim E. Hennink [b], Tina Vermonden[b] and Rick M. Dijkhuizen[a]

[a]Biomedical MR Imaging and Spectroscopy Group, Center for Image Sciences, University Medical Center Utrecht and Utrecht University, Utrecht, the Netherlands, [b]Department of Pharmaceutics, Utrecht Institute for Pharmaceutical Sciences, University Utrecht, Utrecht, the Netherlands, [c]Department of Neurology, University of Kentucky, Lexington, KY, USA

** Corresponding author. Center for Image Sciences, University Medical Center Utrecht, Heidelberglaan 100, 3584 CX Utrecht, the Netherlands. E-mail address: G.A.F.vanTilborg@umcutrecht.nl*

Number of pages: 4

Number of figures: 2

Number of tables: 0

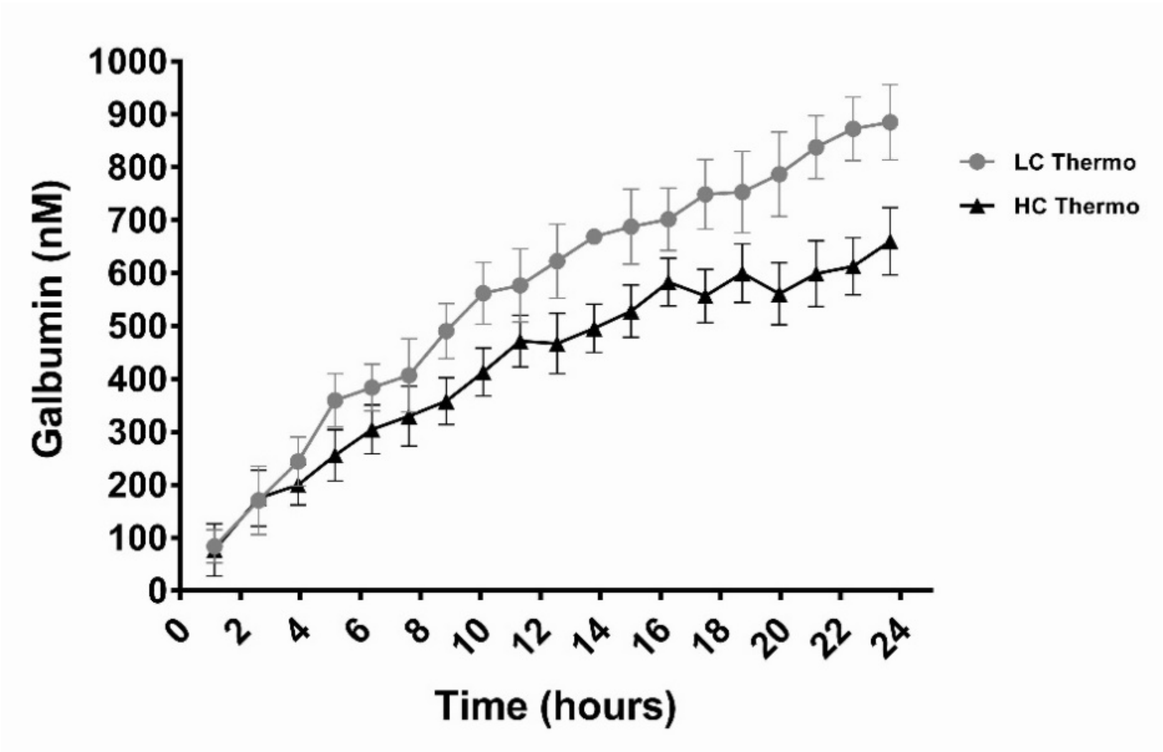

Supplementary Figure 1. Galbumin release from in vitro hydrogel samples at 37 °C over the first 24 hours after gelation. Concentration of Galbumin increases in the PBS supernatant of the corresponding LC Thermo and HC Thermo gel samples. Each point represents the mean value ± SD (n=3).

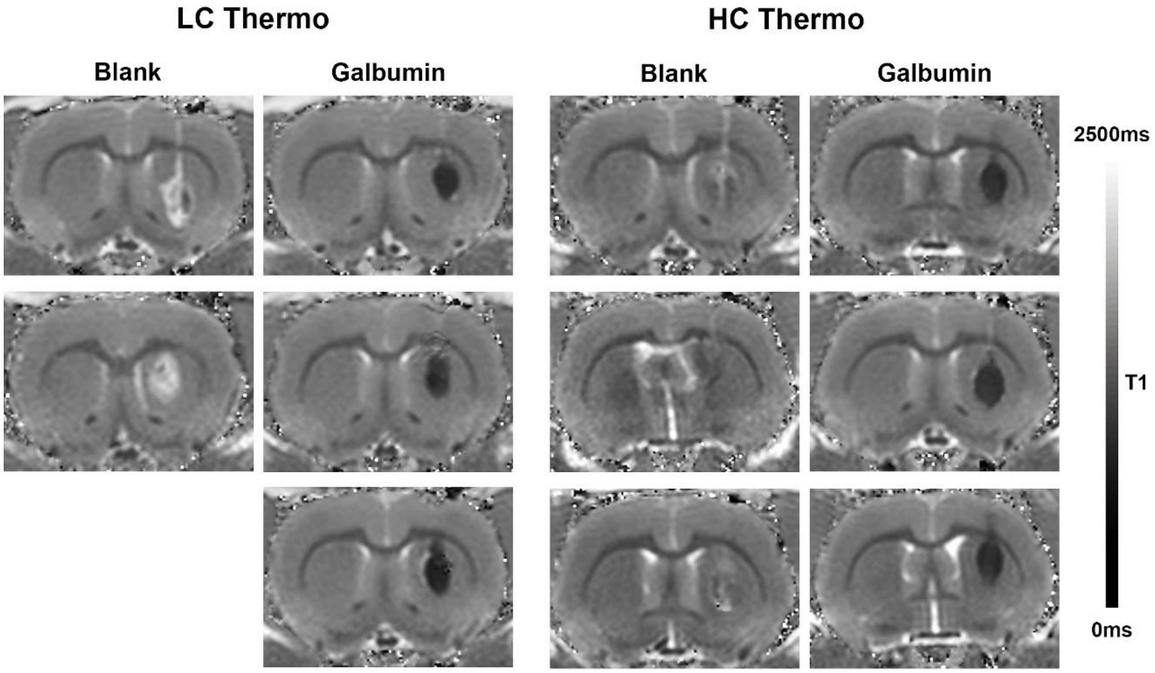

Supplementary Figure 2. T_1 maps of a coronal brain slice for each rat included in the study, acquired directly after stereotaxic injection of 5 µl thermosensitive gel in healthy rat brain. Gels were either non-loaded (blank) or loaded with a protein that was labeled with a T_1-shortening MR contrast agent (Galbumin). Each image shows a brain slice from a different rat (LC Thermo (blank: n=2; Galbumin-loaded: n=3) and HC Thermo (blank: n=3; Galbumin-loaded: n=3)). T_1 maps shown were acquired at the center of the injected volume.

Diffusion coefficients estimation

We estimated the diffusion coefficients using mathematical models for diffusion from a slab of gel in a reservoir system, using equation 7 with cumulative release M_t at 24 hours (38% for LC Thermo versus 25% for HC Thermo) and cumulative release M_∞ after the plateau was reached (71% for LC Thermo versus 50% for HC Thermo) [1]. The diffusion coefficients of Galbumin in LC Thermo and HC Thermo were 5.0×10^{-6} mm^2/s and 4.3×10^{-6} mm^2/s, respectively. For reference, the diffusion of albumin in water is 59×10^{-6}

mm^2/s) [2]. It should be noted that these estimates were based on mathematical modeling for an immersed slab, instead of a cone filled with gel at the bottom of the test tube, covered only from the top. Thus, we may anticipate slight discrepancies in the actual release kinetics.

References

[1] J. Siepmann, F. Siepmann, Modeling of diffusion controlled drug delivery, J. Control. Release, 161 (2012) 351-362.

[2] U. Arunyawongsakorn, C.S. Johnson, Jr., D.A. Gabriel, Tracer diffusion coefficients of proteins by means of holographic relaxation spectroscopy: application to bovine serum albumin, Anal. Biochem., 146 (1985) 265-270.
